# Exploring autistic traits in anorexia: a clinical study

**DOI:** 10.1186/2040-2392-4-44

**Published:** 2013-11-12

**Authors:** Kate Tchanturia, Emma Smith, Felicitas Weineck, Eliz Fidanboylu, Nikola Kern, Janet Treasure, Simon Baron Cohen

**Affiliations:** 1 King’s College London, Institute of Psychiatry, De Crespigny Park, London SE5 8AF, UK; 2 Psychological Medicine Clinical Academic Group, South London and Maudsley NHS Trust, London, UK; 3 King’s College London, Mental Health Studies Programme, Institute of Psychiatry, London, UK; 4 Autism Research Centre, Psychiatry Department, Cambridge University, Cambridge, UK; 5 Cambridgeshire and Peterborough NHS Foundation Trust, Cambridge, UK

**Keywords:** Anorexia nervosa, Autism spectrum conditions, Set-shifting, Detail focus, Theory of mind, Intervention

## Abstract

**Background:**

The objectives of this study were to explore associations between autistic traits and self-reported clinical symptoms in a population with anorexia nervosa (AN). Experimental and self-report evidence reveals similarities between AN and autism spectrum condition (ASC) populations in socio-emotional and cognitive domains; this includes difficulties with empathy, set-shifting and global processing. Focusing on these similarities may lead to better tailored interventions for both conditions.

**Methods:**

A cross-sectional independent-groups design was employed. Participants with AN (*n* = 66) and typical controls (*n* = 66) completed self-report questionnaires including the Short (10-Item) Version Autism Spectrum Quotient (AQ-10) questionnaire (the first time this has been implemented in this population), the Eating Disorder Examination Questionnaire, the Hospital Anxiety and Depression Scale and the Work and Social Adjustment Scale. Group differences and the relationship between autistic traits and other questionnaire measures were investigated.

**Results:**

The AN group had a significantly higher AQ-10 total score and a greater proportion scored above the clinical cut-off than the control group. Seven out of ten AQ-10 items significantly discriminated between groups. In the AN group, levels of autistic traits correlated with a greater self-reported anxiety and depression and a lower ability to maintain close relationships; however, eating disorder symptoms were not associated with autistic traits.

**Conclusions:**

Women with anorexia possess a greater number of autistic traits than typical women. AQ-10 items that discriminated between groups related to ‘bigger picture’ (global) thinking, inflexibility of thinking and problems with social interactions, suggesting that autistic traits may exacerbate factors that maintain the eating disorder rather than cause the eating disorder directly. Using screening instruments may improve understanding of patients’ problems, leading to better tailoring of intervention. We conclude that further investigation of autistic traits in AN could inform new intervention approaches based on joint working between ASC and eating disorder services.

## Background

Anorexia nervosa (AN) is a potentially life-threatening illness associated with high levels of functional and social impairment [1], often with a chronic course [2,3]. Autism spectrum conditions (ASC) and autistic traits are overrepresented in individuals with chronic AN [4,5]; therefore, autistic traits may increase treatment resistance. Similarities between AN and ASC were first observed in the 1980s by Christopher Gillberg. He subsequently proposed that AN should be conceptualized as an empathy disorder on the same spectrum as autism [6]. Thirty years later, this hypothesis is gathering evidence, with reports of shared endophenotypes in AN and ASC [5,7-9], the suggestion that AN may be a female presentation of ASC [7] and work identifying atypical eating behaviours in ASC [10]. Gillberg’s hypothesis was informed by longitudinal studies indicating that ASC is overrepresented in the AN population [11]. In other samples, 16% of teenagers with AN had a premorbid diagnosis of ASC [12] and 23% of adults with AN met the clinical criteria for ASC [13].

Despite differences in presentation, such as a later age of onset in AN [14], a biased sex ratio towards females in AN vs. a biased sex ratio towards males in ASC [15,16], and higher than average IQ in AN [17] vs. more heterogenous IQ levels in ASC [18], empirical studies from the last decade support an overlap in behavioural and cognitive features in AN and ASC. A brief review of relevant work is outlined next.

Deficits in social reciprocity represent part of the diagnostic criteria for ASC [19]. In AN, patients have difficulty recognizing emotional stimuli [14,20] and expressing emotions [21]. In addition, theory of mind inefficiencies have been reported in AN [22] and bulimia nervosa [23]. Self-report measures reveal elevated levels of social anhedonia [24], reduced empathy [25] and impaired relationships and social leisure activities [1,26] in AN. Considering these converging lines of evidence, findings in AN are likely to reflect the difficulties with empathy observed in ASC [27,28], although it should be noted that nearly all psychopathologies are associated with some form of social difficulty and this is not unique to AN.

Similarities have also been noted in the non-social cognitive profiles of AN and ASC, including inefficiencies in set-shifting and global processing. In the autism literature, poor set-shifting, perseveration and repetitive responses in the context of changing environmental demands have been consistently documented [29,30]. Studies investigating set-shifting with large samples of patients with AN have also reported problems in this domain [31,32]; this is supported by a systematic review of cognitive flexibility studies in eating disorders [33]. The authors concluded that people with AN are less able both to adapt to changing rules and to employ strategies flexibly, relative to controls.

With regard to global processing, extensive research reported in the autism literature suggests that individuals with ASC have a superior eye for detail [34,35]. Experimental research describing the same cognitive style in AN is summarized in a systematic review of the literature of AN [36] and in subsequent studies [37-39]. It is important to note that despite similar behavioural outcomes for investigations of central coherence in the ASC and AN literature, recent fMRI studies demonstrated activation in different brain areas when comparing AN and ASC participants during an embedded figure task [40]. This is the first study of its kind and therefore requires further exploration.

Another paradigm employed to investigate commonalities in AN and ASC is the use of self-report questionnaires assessing characteristics associated with ASC, including superior ‘systemizing’ and below average ‘empathizing’ [41,42]. Patients with AN scored significantly higher than controls on the autism spectrum quotient (AQ-10) [43], a measure of autistic traits in adults of average intelligence or above [44]. However, differences on measures of empathizing and systemizing were not observed. When replicated in a larger sample, the scores of participants with AN were significantly different to those of typical controls on all three measures [4].

In summation, the experimental and self-report evidence shows overlapping characteristics in the socio-emotional and non-social cognitive domains in people with AN or ASC. In both conditions, treatment success is limited and it may be that people with comorbid AN and ASC respond particularly poorly to standard treatment programmes for AN. Focusing on the similarities between the two may lead to better tailored interventions for both conditions. In this study we investigated the prevalence of autistic traits in a clinical population receiving treatment for AN in a large national eating disorder service in London. The main aims were to implement a brief screening measure (the AQ-10 [45]) for the first time in a population with AN and to explore associations between autistic traits and eating disorder symptoms, motivation to change, mood and work and social adjustment in a sample of AN and typical women. Based on the outlined literature, we predicted that individuals with AN would score higher on the AQ-10 than controls. No further predictions were made regarding associations between AQ-10 scores and other self-report measures, as this analysis was undertaken on an exploratory basis.

## Method

### Participants

A cross-sectional independent-groups design was employed. Two groups were recruited with a total of *n* = 132 participants. The clinical group consisted of 66 female participants who fulfilled DSM–IV (1994) [46] criteria for AN. A diagnosis of AN was established by a qualified clinician using the Structured Clinical Interview for DSM-IV (SCID) [46] Axis I disorders. Body mass index (BMI; kg/m^2^) was obtained on the day of testing from all participants. Patients for this study were recruited from eating disorder specialist services (50 inpatients; 12 daycare patients; 4 outpatients). Sixty-six typical female control (control) participants were recruited from the local community by advertisement in local libraries, leisure centres, hairdressers and beauty shops, newspapers and online forums.

All participants spoke fluent English. Exclusion criteria for all participants included a history of serious head injury or psychosis. The control participants were screened for additional exclusion criteria on the basis of self-report questions. The additional exclusion criteria included: a family or personal history of psychiatric illness; current use of psychotropic medication; a low (<18) or very high (>26) BMI, and positive answers to screening questions suggestive of eating disorder symptomatology, anxiety or depression. From the original 89 control participants recruited, 23 were excluded from the analysis as they reported a neurological condition (2), were prescribed antidepressants (1), reported answers suggestive of eating disorder symptomatology (2), had a BMI above 27 (6) or below 18 (1) or scored above the recommended clinical cut-off point of 11 on the Hospital Anxiety and Depression Scale (HADS) subscales (11). Informed, written consent was obtained from all participants.

### Measures

#### Demographic variables

All participants were weighed and had their height measured to calculate BMI. Age, ethnicity, educational background and occupational status were also recorded. Participants with AN were asked to record the age at which they were diagnosed with AN, the duration of their illness, and their psychotropic medication status.

#### Autism Spectrum Quotient for Adults (short version; AQ-10)

The AQ-10 [45] was developed from the original 50-item version as a screening tool for clinicians. Responses are on a four-point scale: definitely disagree, slightly disagree, slightly agree and definitely agree. Responses indicating autistic traits score 1, while other responses score zero; hence, a high score corresponds to more autistic traits. Certain questions are reverse scored to prevent response set. A clinical cut-off score of 6 was established from the large scale development and validation study [45], giving sensitivity and specificity values of 0.88 and 0.91, respectively. Internal consistency was high (<0.85).

#### Motivational Ruler (MR)

The MR [47] is a simple Likert scale measuring self-reported importance and ability to change. Higher scores indicate greater importance or ability, respectively. The questions are worded as follows:

1. Importance to change. Ask yourself the following question: How important is it for you to change? What are your desires, reasons and needs for change? What score would you give yourself out of 10?

2. Ability to change. Ask yourself the following question: How confident are you in your ability to change? What score would you give yourself out of 10?

#### Eating Disorder Examination Questionnaire – version 4 (EDE-Q)

The EDE-Q [48] is a 36-item self-report measure of eating disorder symptomatology and behaviours and provides severity scores across four subscales (dietary restraint, weight concern, shape concern, eating concern) and a global score, reflecting overall illness severity. The maximum score on each of the subscales is six, with higher scores indicating greater severity of eating disorder behaviours or beliefs. The EDE-Q has demonstrated good psychometric properties [49].

#### Hospital Anxiety and Depression Scale (HADS)

The HADS [50] is a widely used self-report measure consisting of 14 items that are designed to detect adverse anxiety and depressive states. A cut-off score of 10 is recommended as an indication of ‘diagnosis’ for both scales. In this study, the data from any control participant who scored >11 on either of the HADS subscales was excluded from the final analyses in line with the recommended cut-off [50]. The HADS has good psychometric properties [51].

#### Work and Social Adjustment Scale (WSAS) [52,53]

The WSAS [52-54] is a simple, brief five-item self-report scale designed to measure the degree of functional impairment in the following domains: ability to work; home management; social leisure; private leisure; and ability to form and maintain close relationships. Each item is rated on a 9-point Likert-type scale, ranging from 0 (no impairment) to 8 (very severe impairment). The maximum total score is 40, with higher scores representing greater impairment. The WSAS has demonstrated good internal consistency, test-retest reliability and is sensitive to patients’ perceptions of disorder severity [1].

### Procedure

The study was reviewed and approved by the ethics committee of Kings College London (PNM/12/13-76) for control data collection; patient data were obtained from the clinical audit (National Research Ethics Service (NRES13/LO/0201). All participants were given written information describing the study, and had the opportunity to ask the researcher any questions before providing their informed consent. Participants were also informed of their right to anonymity, and their right to withdraw themselves and their data from the study at any time. Height and weight measurements were taken before participants completed questionnaire packs including demographics, AQ-10, MR, EDE-Q, HADS and WSAS. Finally, participants were provided with written debriefing information. Participants took approximately 15 to 20 minutes to complete all parts of the study.

### Statistical analysis

All data were analyzed using PASW Statistics 20 software. Normality of the data was assessed using Kolmogorov-Smirnov tests, with post-hoc examinations of skewness and kurtosis *z*-scores. Independent *t* tests (or equivalent non-parametric tests for variables that were significantly non-normally distributed) were used to address the hypothesis that the AN group would score significantly higher than the control group on the AQ-10. For the AN group, relationships between the AQ-10 total score and measures of symptomatology and functioning were assessed using Pearson’s product moment correlation coefficients (*r*). Significant correlations were followed up with simple linear regression to explore the predictive value of the AQ-10.

## Results

### Demographic and clinical information

The groups did not differ significantly in terms of their mean age or years in education (Table 1). As predicted, the AN group had a significantly lower BMI than the control group, and reported significantly more severe scores on the EDE-Q, WSAS and HADS scales.

**Table 1:** Between-groups comparisons of demographic and clinical information for anorexia nervosa and control groups

**Variable**	**Anorexia nervosa group *n *** **= 66**^**a **^**(mean (standard deviation))**	**Control group *n *** **= 66**^**a **^**(mean (standard deviation))**	**Test statistic (df)**	***P***
Age	26.35 (8.08)	25.68 (9.74)	*t* (127) = −0.42	0.67
Years in education	15.08 (2.16)	15.78 (3.57)	*t* (107) = 1.12	0.27
Body mass index (kg/m^2^)	14.90 (2.13)	21.78 (2.46)	*t* (124) = 16.7	0.001*
Age of onset	15.52 (5.37)	-	-	
Duration of illness	10.59 (6.66)	-	-	
EDE-Q: global	3.84 (1.57)	1.14 (0.98)	*U* = 411	0.001*
			*Z* = −8.04	
EDE-Q: dietary restraint	3.68 (1.88)	1.08 (1.19)	*U* = 593	0.001*
			*Z* = −7.24	
EDE-Q: eating concern	3.35 (1.61)	0.48 (0.60)	*U* = 213	0.001*
			*Z* = −8.97	
EDE-Q: weight concern	3.90 (1.73)	1.23 (1.24)	*U* = 518	0.001*
			*Z* = −7.57	
EDE-Q: shape concern	4.43 (1.62)	1.79 (1.38)	*t* (130) = −10.07	0.001*
HADS: anxiety	13.82 (4.58)	5.52 (3.07)	*U* = 211.5	0.001*
			*Z* = −7.34	
HADS: depression	10.80 (4.58)	1.99 (2.26)	*U* = 114	0.001*
			*Z* = −7.98	
WSAS total score	28.46 (8.53)	0.80 (3.05)	*U* = 18	0.001*
			*Z* = −10.12	

*P < 0.005 (corrected for multiple comparisons); ^a^In line with statisticians’ recommendations, values are reported for available data where this does not fall below 20% missing. EDE-Q, Eating Disorder Examination Questionnaire; HADS, Hospital Anxiety and Depression Scale; WSAS, Work and Social Adjustment Scale.

### Internal consistency of questionnaires

The internal consistency of the EDE-Q, the WSAS, the AQ-10 and the HADS was assessed by standardized Cronbach’s alpha, alpha if item deleted, inter-item, and item-total correlation coefficients, after data collection. Reverse scored items were recoded before the reliability analysis. Cronbach’s alpha indicated acceptable reliability for all measures: 0.85 for the EDE-Q, 0.97 for the WSAS, 0.76 for the AQ-10 and 0.94 for the HADS.

### Scores on the AQ-10

**Figure 1:**
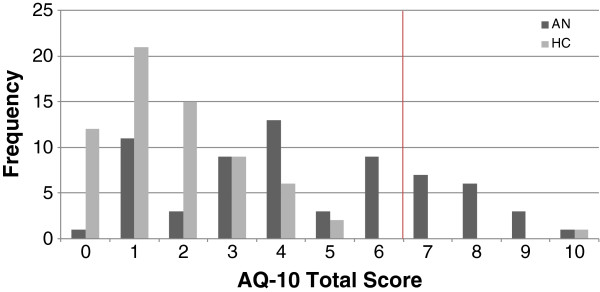
**Proportions of AQ-10 total score in anorexia nervosa and control groups.** Vertical line indicates clinical cut-off score of 6. AN, anorexia nervosa; HC, healthy control.

The AN group had a significantly higher AQ-10 total score (*mean* = 4.53, *standard deviation* = 2.56) than the control group (*mean* = 1.85, *standard deviation* = 1.68), which was associated with a very large effect size, *U* (131) = 846; *Z* = − 6.14, *P =* .000*, d* = 1.24. Significant differences between the AN and control groups were revealed for raw scores on seven out of the ten items of the AQ-10, applying the Bonferroni correction for multiple comparisons (Table 2). The frequency of high scorers was also significantly different between the AN and control groups; more people in the AN group (25.8%) than the control group (1.5%) scored above the clinical cut-off score of 6, *χ*^2^ (1, *N =* 132) = 16.47, *P* = .001, as illustrated in Figure 1.

**Table 2:** Between-groups comparisons of raw scores on AQ-10 individual items for anorexia nervosa and control groups

**AQ-10 item**	**Anorexia nervosa *n *** **= 66 (mean (standard deviation))**	**Control *n *** **= 66 (mean (standard deviation))**	**Test statistic (df)**	***P***	**Effect size**
Noticing small sounds	2.66 (1.07)	2.12 (1.04)	*t* (130) = −2.96	0.004*	*d* = 0.52
Detail focus	2.97 (0.86)	2.07 (0.86)	*t* (130) = −5.96	0.001**	*d* = −1.05
Difficulty multitasking	2.48 (0.99)	1.88 (0.73)	*U* = 1420	0.001**	*d* = 0.69
			*Z* = −3.65		
Difficulty coping with interruptions	2.69 (0.99)	2.04 (0.77)	*U* = 1366.5	0.001**	*d* = 0.73
			*Z* = −3.9		
Difficulty reading between the lines	2.29 (1.08)	1.68 (0.68)	*U* = 1505	0.001**	*d* = 0.68
			Z = −3.23		
Failure to notice boredom in others	1.77 (0.97)	1.48 (0.61)	*U* = 1923	0.195	*d* = 0.36
			*Z* = −1.29		
Difficulty working out characters’ intentions	2.18 (0.86)	1.79 (0.89)	*t* (130) = −2.56	0.01	*d* = −0.45
Collecting information	2.06 (1.10)	1.36 (0.62)	*U* = 1611	0.006*	*d* = 0.78
			*Z* = −2.73		
Difficulty reading faces	2.09 (0.97)	1.86 (0.76)	*t* (130) = −1.50	0.14	*d* = −0.26
Difficulty working out others’ intentions	2.64 (0.94)	1.98 (0.90)	*t* (130) = −4.06	0.001**	*d* = −0.71

*P < 0.01 (corrected for multiple comparisons); **P < 0.001. AQ-10, autism spectrum quotient.

### Relationship of AQ-10 to clinical measures

A Pearson product moment coefficient was computed to test associations between AQ-10 score and questionnaire measures for the AN group (Table 3). A conservative *P* value of 0.01 was employed to adjust for multiple comparisons. HADS anxiety and depression scores and scores on the ‘ability to maintain relationships’ subscale of the WSAS were significantly positively correlated with total AQ-10 scores, meaning that the higher the number of autistic characteristics reported, the more problems individuals seem to experience in work and social adjustment domains and mood. To explore this association further, a series of simple linear regression analyses were conducted. AQ-10 total scores significantly predicted self-reported difficulty in forming and maintaining relationships, *b* = 0.278, *t* (63) = 2.99, *P* = 0.004 and explained 12.5% of the variance in this domain (adjusted *R*^2^ = 0.111), *F* (1, 63) = 8.99, *P* = 0.004. AQ-10 total scores also significantly predicted anxiety scores, *b* = 0.805, *t* (39) = 2.97, *P* = 0.005 and explained 18.4% of the variance in anxiety scores (adjusted *R*^2^ = 0.163), *F* (1, 39) = 8.80, *P* = 0.005. AQ-10 total scores further significantly predicted depression scores, *b* = 0.777, *t* (39) = 2.84, *P* = 0.007 and explained 17.1% of the variance in depression scores (adjusted *R*^2^ = 0.150), *F* (1, 39) = 8.05, *P* = 0.007.

**Table 3:** Correlations between AQ-10 total score and symptomatology

**Variable**	**Anorexia nervosa**	
	**1**	***P***
EDE-Q: Global	*r* (66) = .12	0.35
EDE-Q: Dietary restraint	*r* (66) = .02	0.89
EDE-Q: Eating concern	*r* (66) = .18	0.15
EDE-Q: Weight concern	*r* (66) = .15	0.22
EDE-Q: Shape concern	*r* (66) = .09	0.48
HADS: Anxiety	*r* (41) = .43	0.005*
HADS: Depression	*r* (41) = .41	0.007*
WSAS: Total	*r* (65) = .29	0.02
WSAS: Work	*r* (65) = .18	0.14
WSAS: Home management	*r* (65) = .15	0.23
WSAS: Social leisure	*r* (65) = .24	0.08
WSAS: Private leisure	*r* (65) = .20	0.05
WSAS: Relationships	*r* (65) = .35	0.004*
Motivation: Importance	*r* (55) = -.05	0.73
Motivation: Ability	*r* (56) = *-.28*	0.04

*P < 0.01 (corrected for multiple comparisons). AQ-10, autism spectrum quotient; EDE-Q, Eating Disorder Examination Questionnaire; HADS, Hospital Anxiety and Depression Scale; Motivation, Motivational Ruler; WSAS, Work and Social Adjustment Scale.

## Discussion

This study set out to investigate autistic traits in individuals with AN. Individuals with AN scored significantly higher on the AQ-10 than typical control participants. Furthermore, analysis of responses to individual items of this scale revealed significant differences for the majority of questions. This supports the hypothesis that women with anorexia possess a greater number of autistic traits than typical women and replicates the findings of previous studies [4,44]. In addition, this study goes beyond the existing literature as the first study using the AQ-10 in a population with AN. Using the clinical cut-off recommended by the scale’s authors, more than a quarter of the AN group obtained a score high enough to warrant a referral to an ASC assessment service (referrals were made in these instances), closely reflecting the frequency of patients meeting full clinical criteria in an adult AN population [13]. In contrast, less than 2% of the control participants scored over the cut-off point.

Following on from these results, an exploratory analysis of how these elevated levels of autistic traits were related with other self-reported symptoms in the AN group revealed a strong correlation with mood, including both anxiety and depression, and the ability to maintain close relationships. Regression analyses showed a predictive role of the AQ-10 total score, indicating that higher levels of autistic traits are associated with feeling anxious and depressed and that this might explain difficulties in interpersonal interactions for people with AN [1]. It is interesting to note that significant correlations were not observed between autistic traits and eating disorder symptoms. It is important to consider whether the AQ-10 is in fact measuring mood symptoms rather than autistic traits, a situation that would threaten the validity of this instrument. Examining the individual items reveals that only certain questions may directly relate to emotional problems (for example, difficulty multitasking and coping with interruptions relates to poor concentration) with a greater number tapping into other cognitive or socio-emotional domains. This would suggest that symptoms of anxiety and depression might increase scores slightly; however, the range of questions appears to reflect typical autistic traits and therefore have good face validity. How can the significantly higher AQ-10 scores in individuals with AN be reconciled with a failure to demonstrate a direct link between autistic traits and disordered eating? It could be speculated that autistic traits exacerbate ‘maintaining factors’ for eating disorder, such as cognitive rigidity, low mood, low motivation or a lack of social skill, with the latter two factors relating to the significant correlations reported here, rather than directly causing the eating disorder behaviours. In a recent study, eating disorders (AN and bulimia nervosa), anxiety disorders and depression accounted for a large portion of difficulties with social functioning [53]. Although the outcome measure was somewhat different in this study, it suggests that eating symptoms, as well as mood, are expected to be associated with autistic traits falling within the social domain.

Nevertheless, the current study along with previous research indicates a significant trend: people with AN are more likely to show autistic traits. Several factors might play a part in this. For example, starvation has been shown to reduce the quality of interpersonal relationships, interest in other people, and libido [55]. Secondly, a detailed assessment of the items on the AQ-10 revealed that seven out of the ten items discriminated between patients with AN and control participants. These items tap into problems with ‘bigger picture’ (global) thinking (item 1, 2 and 7); inflexibility of thinking (item 3 and 4) and problems with social interactions (item 5 and 10).

In the adult literature, the presence of difficulties in these three domains in the AN population has received empirical and experimental support. For example, problems with abstract thinking are reported in a systematic review conducted by Lopez *et al*. [36] and have been shown to be experienced by AN patients in experimental studies [38,39]. In addition, problems with flexibility of thinking are reported in the systematic review [33] and large data-based studies of Tchanturia *et al*. [31,32]. It is also notable that social anhedonia [1] and problems with social cognition [5,20] have been widely reported in the literature.

The items that did not significantly discriminate between patients with AN and controls appear to tap into theory of mind abilities, including difficulty reading faces and working out characters’ intentions. Despite the literature described (for example, [14,20-22]), this is in accordance with a psychometric study reporting that both AN and ASC groups demonstrated a need for sameness; however, other core features of autism, such as difficulty empathizing, were not seen in the AN group [56].

### Limitations

A main aim of the study was to test the AQ-10 in an eating disordered population; therefore, the primary outcomes were self-reported. For future studies, additional clinical interviews and experimental (performance-based task) designs could improve the validity and reliability of findings. The AN group consisted of inpatients, daycare patients and outpatients; this may have introduced heterogeneity into the data, owing to varying levels of illness severity. However, all patients met the DSM–IV criteria for AN and were receiving treatment. Finally, it was not possible to recruit a recovered AN group; this may be useful in exploring whether elevated levels of autistic traits are present in the recovered form of this eating disorder diagnostic group as well as the acute state. This is an important area to investigate, as a recent study has reported that difficulties with social cognition and communication were less prominent in a recovered AN group [57]. This raises the possibility that high scores on measures of autistic traits could be, in part, a consequence rather than a cause of AN.

### Clinical implications

Exploring links between ASC and AN can provide important insights into the aetiology of both conditions. In terms of clinical implications, women are a challenging group for ASC professionals to diagnose [58], while AN is the most challenging condition to treat on the eating disorder spectrum [59]. Brief screening instruments, such as the AQ-10, provide helpful and easy tools that facilitate better understanding of the patient’s problems and may lead to better tailoring of intervention. It is possible that women with AN who have high scores on the AQ-10 might benefit more from cognitive therapies than from other psychological or pharmacological treatments that do not directly address problems with set-shifting, bigger picture thinking or socio-emotional deficits. Existing module-based treatment packages (for example, cognitive remediation therapy [60,61] and cognitive remediation and emotion skills therapy [62]) provide targeted interventions with which to address ASC symptoms in a population with AN. Further research is required to investigate the effectiveness of these intervention packages in relation to ASC symptomatology.

## Conclusions

Individuals with AN scored higher than controls on the short version AQ-10, a measure that has not previously been used with this population. Levels of autistic traits correlated with self-reported mood and an ability to maintain close relationships; however, eating disorder symptoms were not associated with autistic traits. Further investigation of these findings would benefit from a comparison group of participants with an ASC diagnosis. The role of autistic traits in the presentation of eating disordered behaviour requires further investigation and has the potential to inform new intervention approaches based on joint working between ASC and eating disorder services.

## Abbreviations

AN: Anorexia nervosa; AQ-10: Autism spectrum quotient; ASC: Autism spectrum condition; BMI: Body mass index; EDE-Q: Eating disorder examination questionnaire; HADS: Hospital anxiety and depression scale; IQ: Intelligence quotient; MR: Motivational ruler; WSAS: Work and social adjustment scale.

## Competing interests

The authors declare that they have no competing interests.

## Authors’ contributions

KT conceived the study, participated in its design, coordination, data collection and drafted and edited the manuscript. ES, FW and EF contributed to the design of the study, data collection and statistical analyses and helped draft the manuscript. NK and JT participated in the study design. SBC helped to draft and finalize the manuscript. All authors read and approved the final manuscript.
